# Indicators for Increased Likelihood of Epidemic Kaposi Sarcoma Progression After Antiretroviral Therapy Initiation

**DOI:** 10.1089/aid.2025.0007

**Published:** 2025-07-01

**Authors:** David J. Nolan, Gary B. Fogel, Jonathan DaRoza, Rebecca Rose, Paige M. Bracci, Susanna L. Lamers, Michael S. McGrath

**Affiliations:** 1 Bioinfoexperts, LLC, Thibodaux, Louisiana, USA.; 2 Natural Selection, Inc., San Diego, California, USA.; 3 Department of Medicine, The University of California at San Francisco, San Francisco, California, USA.; 4 Department of Epidemiology and Biostatistics, University of California, San Francisco, San Francisco, California, USA.

**Keywords:** Kaposi sarcoma, KSHV, HIV, antiretroviral therapy

## Abstract

Kaposi sarcoma (KS) is a common malignancy for people living with HIV (PLWH), despite antiretroviral therapy (ART). Curiously, even with improved CD4^+^ T-cell counts and low viral loads following ART, some PLWH with KS may still experience KS progression or even death and require adjuvant chemotherapy to manage their KS. The factors associated with persistent or unresponsive KS after ART initiation remain poorly characterized, and biomarkers to identify patients at risk of KS progression are needed, particularly in resource-limited areas where access to chemotherapy is limited. Here we analyzed baseline KS tumor biopsies from PLWH with KS who required chemotherapy due to unresolved KS after ART initiation and those who did not require chemotherapy after ART initiation. By examining participant metadata and viral copy number for Kaposi sarcoma-associated herpesvirus (KSHV), HIV, cytomegalovirus, and Epstein-Barr virus and KSHV gene expression in the tumor biopsies prior to ART initiation, we identified a model of factors associated with KS progression after ART initiation, including biological sex, age, and the log ratio of KSHV/HIV copy number in the tumor. We believe that the ratio of KSHV/HIV may be linked to the cell types that each virus infects, and future work exploring the relationship between tumor and immune cells in the baseline tumors is planned. Innovation would be necessary to reduce costs and simplify the viral quantification assays, enabling the translation of these findings into routine clinical care, particularly in resource-limited settings.

## Introduction

Kaposi sarcoma (KS) is caused by Kaposi sarcoma-associated herpesvirus (KSHV), also known as human gammaherpesvirus 8 (HHV-8),^1^ and is characterized by highly vascularized, darkly colored skin lesions. Epidemic KS, classified epidemiologically by coinfection with human immunodeficiency virus (HIV), displays aggressive clinical features.^2^ Antiretroviral therapy (ART) has significantly reduced the burden of KS for people living with HIV (PLWH); however, KS remains one of the most common malignancies in PLWH, even with effective ART.^3^ Although ART alone can effectively treat PLWH with limited cutaneous disease, KS does not resolve in 20%–50% of PLWH undergoing ART, and they can progress to death despite improved CD4^+^ T-cell counts and low viral loads.^4^ This is especially true in sub-Saharan Africa, where seroprevalence of both viruses is highest worldwide,^5,6^ and chemotherapy may not be readily available.^7,8^ Overall, the combination of factors that lead to persistent or unresponsive KS after ART initiation remains ill-defined,^3,4^ and biomarkers to target adjuvant chemotherapy among those at risk of KS progression after ART initiation^9^ are needed to identify at-risk patients and to improve the prognosis of PLWH diagnosed with KS. Recent studies have presented intriguing candidates [e.g., high plasma KSHV copy number, elevated C-reactive protein (CRP), and IL-10 serum biomarkers] for predicting KS progression in PLWH on ART,^10,11^ but given the small numbers of study participants in these studies they require validation in larger, more heterogeneous populations.

The complexity of KS tumors makes it difficult to identify features that distinguish persons who would experience KS resolution or progression after initiating ART. Unlike classical cancer metastasis, KS tumors are polyclonal, with individual tumors arising independently.^12^ KS tumors are also histologically complex, with interactions among various cell types. Spindle cells, the defining abnormal elongated cells of KS tumors, have markers of endothelial and lymphatic origin and are infected with KSHV.^12^ T cells, B cells, and macrophages are also present, and subsets of these cells may be HIV-infected.^13,14^ The KSHV DNA genome persists as an episome within infected cells, at one or two copies on average^15^ and is necessary for developing and maintaining KS tumors.^16^ The KSHV viral cycle is divided into two phases: a replicative or lytic phase and a latency phase characterized by the expression of a small number of genes, including LANA-1, v-FLIP, and v-cyclin. In both the lytic and latent phases, KSHV encodes oncogenic proteins that modulate gene expression in the cells of the KS tumor to produce features found in most cancers, including angiogenesis, inflammation, proliferation, inhibition of apoptosis, and immune escape.^17^

In this study, we focused on tumor biomarkers associated with viral infection. Tumor punch biopsies were obtained from PLWH diagnosed with KS who participated in a clinical-epidemiology study investigating the effect of drug regimens on outcomes.^18^ We divided the participants into two groups: progressors, defined as having unresolved KS that required chemotherapy after ART initiation, and responders, whose KS did not require chemotherapy in addition to ART. We analyzed DNA and RNA from KS tumor punch biopsies collected before ART initiation for viral copy number of KSHV, HIV, cytomegalovirus (CMV), and Epstein-Barr virus (EBV), as well as KSHV gene expression. These measurements were analyzed independently and in combination with other viral (i.e., subtype and genotype) and demographic factors to develop a model of factors associated with KS progression after ART initiation. With success, such a model could assist in focusing early chemotherapy on the PLWH in resource-limited settings most at risk of KS progression after ART initiation.

## Methods

### Outcomes cohort

**FIG. 1.**
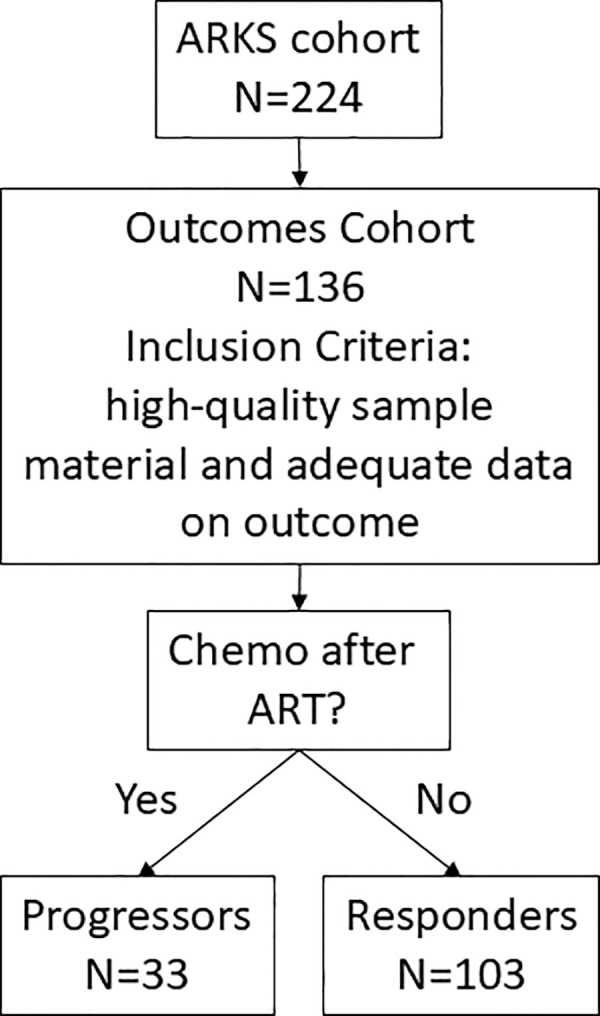
Outcomes cohort flowchart. The original ARKS cohort enrolled 224 Uganda PLWH from 2007 to 2011 who had limited-stage KS and no history of prior ART and chemotherapy. The participants were followed for 12 months and initially treated with ART alone and only given chemotherapy if necessary to treat progressive disease. After the ARKS study was completed, the ACSR acquired the ARKS samples. A subset of cases was selected to form the outcomes cohort (*n* = 136) based on the availability of high-quality sample material from before and after ART, and adequate metadata about the participants and their outcomes. KS progression status was defined in the outcomes cohort as: progressors, those who required chemotherapy to control their KS progression while on ART, and responders, those who did not receive chemotherapy. ACSR, AIDS and Cancer Specimen Resource; ARKS, Antiretrovirals in Kaposi Sarcoma; KS, Kaposi sarcoma; PLWH, people living with HIV.

Biospecimens were obtained from the Antiretrovirals in Kaposi Sarcoma (ARKS) study,^18^ a randomized, single-blinded trial in Uganda from 2007 to 2011 that compared the effect of two ART regimens. Study participants were ART-naïve PLWH (≥18 years old) with newly diagnosed KS who were deemed to have no functional impairments that necessitated immediate chemotherapy. Participants with active, untreated opportunistic infection or other malignancies were excluded. Participants were initially treated with ART alone and followed for 48 weeks. Results showed no significant difference in the primary outcome, indication for chemotherapy or death (whichever came first), between the ART-regimen groups; thus, all participants were pooled for the current analysis. ARKS biospecimens were donated to and banked at the AIDS and Cancer Specimen Resource (ACSR)^19^ and were obtained for this study after independent approval via the ACSR’s letter of intent process. A subset of cases was selected based on the availability, quantity, and quality of biospecimens to form the outcomes cohort, defined as progressors (*n* = 33), those patients who needed chemotherapy to resolve KS while taking ART, and responders (*n* = 103), those who did not require chemotherapy (Fig. 1).

### Nucleic acids

DNA and RNA were prepared from a KS lesion punch biopsy specimen from each outcomes cohort participant before ART initiation. Each punch biopsy specimen was preserved in RNA later at the time of collection and stored at −80°C. DNA and total RNA were extracted at George Washington University using a novel and highly efficient approach, resulting in high-quality nucleic acids.^20^ The requested nucleic acids were shipped on dry ice and stored upon arrival at −80°C until needed.

### Restriction enzyme digestion

Tumor DNA (625 ng) was digested with restriction enzyme *Ban*II (New England Biolabs R0119S) following the manufacturer’s guidelines. Briefly, DNA was diluted into 25 µL 1X CutSmart Buffer containing 50 units of *Ban*II enzyme and incubated for 1 h at 37°C. The resulting digested DNA (25 ng/µL) was stored at −20°C until needed for assays.

### Reverse transcription

Tumor RNA was diluted to 30 ng/µL before cDNA conversion. All samples’ RNA was converted to cDNA in the same reaction volume to minimize batch effects in the subsequent quantification assays. RNA was reverse transcribed using Invitrogen SuperScript IV First-Strand Synthesis System (ThermoFisher Scientific 18091050) following the manufacturer’s recommendations with one exception: a 1:5 mixture of the Oligo(dT)_20_ (50 μM) and random hexamers (50 ng/μL) was prepared and added to the RNA prior to reverse transcription. After the conversion, cDNA was stored at −20°C until use.

### Gene quantification assays

Various primers and probes were used for this work (Supplementary Table S1, Primers and Probes), including one commercial assay for human *IL-10* quantification. Quantitative PCR TaqMan assays were performed on tumor DNA for the KSHV orf25 gene^21,22^ and the human RNAse P (*RPP30*) gene. CMV and EBV quantification was performed using a previously published protocol,^23^ which provides detailed instructions on multiplexing two assays in one reaction using FAM and HEX dyes. The HIV assay used targets the HIV-1 long terminal repeat region^24^ and was previously tested and shown to perform well using several reference collections, including known HIV subtypes and recombinants.^25^ Gene expression assays were used on tumor RNA that had been converted to cDNA and included human actin,^26^ *c-MYC*,^27^ *IL-10* (ThermoFisher Scientific Hs00961622_m1), and several KSHV genes including orf73 LANA^27^ as well as orf71 v-FLIP, orf72 vCyclin, orf74 vGPCR, and K10.5/K10.6 vIRF3.^26^

### Droplet digital PCR

Digested tumor DNA (100 ng) was used in each droplet digital PCR (ddPCR) assay. The *RPP30* and KSHV orf25 assays were multiplexed together, and the CMV and EBV assays were multiplexed in a different reaction. Given the high expression for actin and LANA in the RNA from baseline tumor biopsies, we assayed 1uL of a 1:10 cDNA dilution (corresponding to 1.66 ng of RNA) in each reaction. For *c-MYC* and *IL-10*, as well as the other KSHV genes, we assayed 5uL of undiluted cDNA, corresponding to 83.3 ng RNA. All reactions followed the same general ddPCR cycling protocol but with differing annealing temperatures: 95°C for 10 min (denature), followed by 50 cycles of 95°C for 30 s (denature), 60°C-54°C for 60 s (annealing and extension), with a final 98°C step and a 4°C hold at the end. All assay reactions were prepared using Bio-Rad ddPCR Supermix for Probes (Bio-Rad 1863924) in a biosafety cabinet. The droplets were prepared on an automated Bio-Rad QX200 Droplet Generator, and then cycled in a Bio-Rad C1000 thermocycler. The cycled reactions were processed on the Bio-Rad QX200 Droplet Reader, and the output was analyzed using the Bio-Rad QuantaSoft Analysis Pro software. The DNA quantification results were expressed as the copy number of the viruses per 100,000 tumor cell equivalents, using the *RPP30* gene for normalization, given there are two copies of *RPP30* in each cell. For the RNA expression results, we used the actin gene expression to normalize the values for the expression of other genes, as well as LANA and KSHV copy numbers for the normalization of KSHV gene expression as a comparison. Welch’s *t*-test, or unequal variances *t*-test, was used to test for associations between viral copy number or gene expression and outcomes group (progressor or responder).

### HIV subtyping

To determine HIV subtype, tumor DNA was PCR amplified using *env-nef* primers in a limiting-dilution single genome sequencing (SGS) format using previously published methods.^28^ Briefly, 1 µL digested DNA (25 ng) was diluted 1:12 and assayed in 12 SGS nested PCR reactions to look for ∼3.6 Kb bands on a 1% TAE agarose gel. If >25% positive reactions were found, DNA was further diluted until ≤3 positive reactions were present. If no positives were found, the remaining digested DNA was utilized until a positive reaction occurred or the digested DNA was exhausted. Positive PCR reactions were sequenced using the amplification primers and a suite of internal sequencing primers at Azenta. Chromatograms were aligned using MAFFT V7.450^29^ implemented in Geneious Prime, and consensus sequences were extracted after manual optimization of chromatogram alignment and base calling. Subtyping of consensus *nef* sequences was conducted using COMET.^30^ Sequences defined as “unassigned” by COMET underwent further analysis with Simplot V3.5.1^31^ for evidence of recombination between HIV subtypes using previously published methods.^32^

### KSHV orfK1 gene subtyping

The K1 gene has been used by other groups extensively to genotype KSHV,^33,34^ and genotype-specific outcomes have been postulated or reported.^35,36^ To determine KSHV genotype, digested DNA was PCR amplified using previously published methods^37^ with primers for the orfK1 sequence (∼870 bp). Briefly, semi-nested PCR reactions were performed in a 25 μL reaction mixture containing ∼50 ng of DNA template and 1X Platinum II Hot-Start Green PCR Master Mix (Invitrogen Life Technologies 14001014). The PCR thermal conditions for the first and second rounds were as follows: initial 3 min denaturation at 95°C; 45 cycles of 95°C for 30 s, 61.5°C for 50 s, and 72°C for 1 min; and a final extension at 72°C for 3 min. PCR products were visualized on 1.5% agarose gels stained with ethidium bromide. PCR products were sequenced at Azenta using second-round amplification primers. Resulting chromatograms were assembled, and consensus sequences were extracted and aligned using Geneious Prime. Consensus orfK1 sequences were aligned with 59 complete KSHV orfK1 reference sequences from the orfK1 genotypes (A–F) for analysis, similar to a genotyping methodology already published.^33^ A phylogenetic tree was inferred using IQ-TREE2.^38^ The model selection for each gene was performed with ModelFinder^39^ implemented in IQ-TREE2 (-MFP option). Ultrafast bootstrap^40^ and SH-like approximate likelihood ratio test (SH-aLRT) were calculated (options -bb 5000 and -alrt 5000) to assess node support. FigTree (http://tree.bio.ed.ac.uk/software/figtree/) was used to view the phylogeny and determine KSHV genotype. Once the sequences were assigned KSHV genotypes, a chi-squared test was used to determine if a subtype was associated with patient outcome.

### Statistical assessments and multivariable logistic regressions

Statistical assessments were made using JMP 13.2.1. (SAS Institute, Inc.) and visualized as boxplots and analyzed using chi-square or Student’s *t*-tests as appropriate. Normality tests indicated that the ratio of log-transformed KSHV to HIV was near normal, so Student’s *t*-test was used to determine significance, although small sample sizes in general make the determination of true normality difficult. Multivariable logistic regression analyses were also conducted using JMP 13.2.1. The performance of factors included in the logistic model was evaluated using receiver operating characteristic curve methods expressed as minimizing the area under the curve (AUC).

## Results

### Sex and age

There was a total of 55 women and 81 men included in the outcomes cohort. A significantly and disproportionately higher proportion of men were progressors (*n* = 25 progressors, *n* = 56 responders) compared to women (*n* = 8 progressors, *n* = 47 responders; *p* = .03). The age of the participants was evaluated independently (Supplementary Table S2) but a significant trend was not found comparing progressors with responders (χ^2^, *p* = .74).

### HIV subtyping

HIV *nef* sequences were generated from baseline tumor biopsy DNA for 112/136 samples, and HIV subtypes were assigned for 102/112 samples with *nef* sequences (Table 1, panel A). Sequences from ten participants were classified as “unassigned” and were likely cases of a recombinant form whose actual classification was ambiguous.^30^ Compared with samples with *nef* sequences, those for which an HIV *nef* sequence could not be generated (*n* = 24, 17.6%) had a significantly lower HIV copy number in the tumor DNA (*t*-test, *p* < .001). No statistically significant difference was found in the HIV subtype assignments by outcome group (χ^2^, *p* = .55).

**Table 1.** HIV *nef* Subtype and KSHV K1 Genotype

Panel A			Panel B		
HIV *nef* subtype	Responders (*n*)	Progressors (*n*)	KSHV K1 genotype	Responders (*n*)	Progressors (*n*)
A1	51	14	A	44	17
D	27	7	B	16	6
C	1	1	C	14	2
Recombinant	7	4	F	0	1
No sequence	17	7	No sequence	29	7

KSHV, Kaposi sarcoma-associated herpesvirus.

### KSHV genotyping

KSHV orfK1 sequences were generated from baseline tumor biopsy DNA for 100/136 samples (Table 1, panel B). Based on their placement in a phylogeny with other orfK1 reference sequences (Supplementary Fig. S1), we identified the following genotypes: responders A = 44, B = 16, C = 14, and 29 with no sequence; progressors A = 17, B = 6, C = 2, F = 1, and 7 with no sequence obtained. KSHV genotype was not associated with outcome group (χ^2^, *p* = .44).

### Viral DNA copy number

**FIG. 2.**
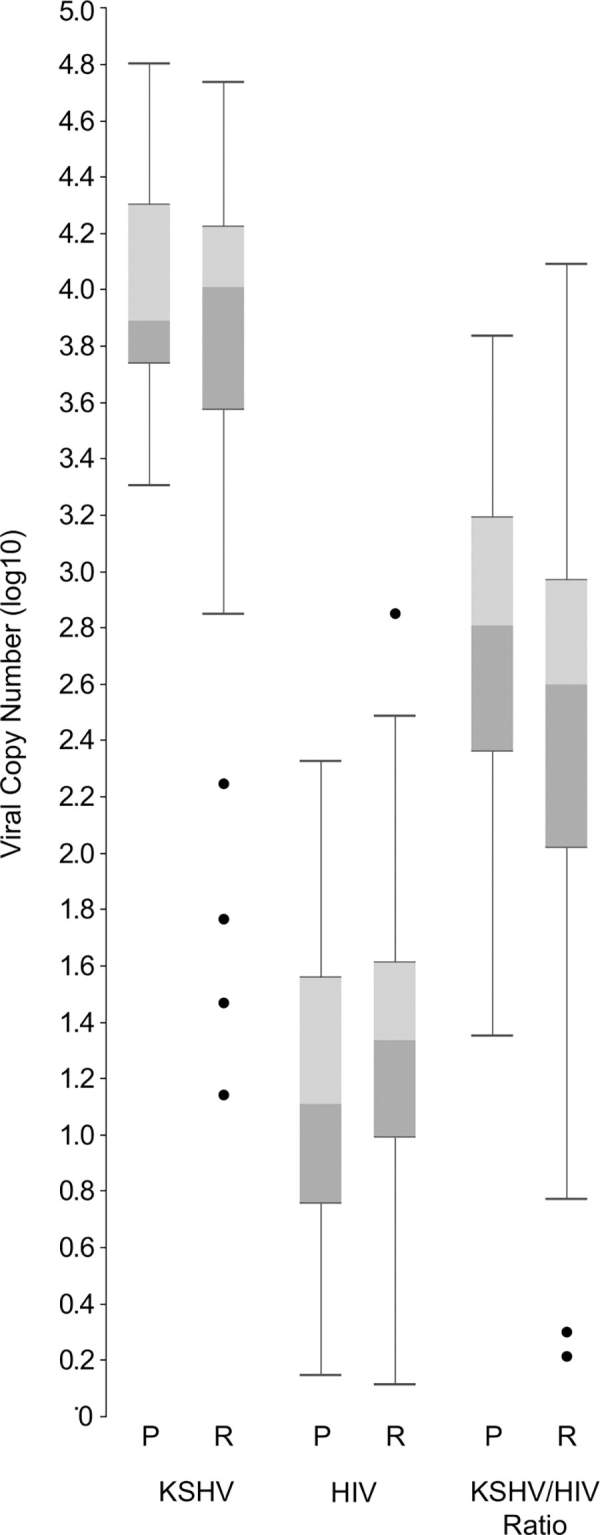
Viral copy number. The log10 viral copy number of KSHV and HIV found in total genomic DNA extracted from KS tumor biopsies was determined using ddPCR. Viral copy number was calculated as copies per 1 × 10^6^ tumor cells using the copy number of the human RNAse P gene, where each cell contains two copies of the RNAse P gene. KS progression status was not associated with copy numbers for KSHV (*t*-test, *p* = .09) or for HIV (*t*-test, *p* = .14). However, by calculating the log10 ratio of KSHV to HIV, a statistically significant trend was found whereby progressors have comparatively higher numbers of KSHV relative to HIV in tumor DNA (*t*-test, *p* = .02). ddPCR, droplet digital PCR; KSHV, Kaposi sarcoma-associated herpesvirus.

KSHV DNA was detected in all tumor samples, while HIV was detected in all except one (KPA5123). CMV and EBV were detected in the tumor DNA infrequently (Supplementary Table S3). Given the large ranges associated with KSHV and HIV copy numbers, log ranges offer improved granularity when comparing the copy number of one virus to another (Fig. 2). Neither KSHV nor HIV copy numbers were associated with outcome group (*t*-test, *p* = .09 and *p* = .14, respectively). However, analysis of the ratio of log-transformed KSHV to HIV showed higher ratios in progressors compared to responders (*t*-test, *p* = .02).

### KSHV gene expression

Actin expression was detected in 134/136 samples. In two samples, actin was either not detected at all (KPA5027) or had a low signal (KPA6480); these two cases were removed from further analysis. KSHV latent genes orf73 LANA (*M* = 6712.74, *SD* = 15233.85) had measurable expression in all 134 cases, whereas orf72 vCyclin (*M* = 235.34, *SD* = 498.42) was undetectable in four cases (1 progressor, 3 responders). Conversely, KSHV latent gene orf71 v-FLIP (*M* = 843.28, *SD* = 1466.12) was undetectable in 43% of cases, and another orf71 assay^41^ was used to confirm these results (data not shown). KSHV vIRF3 (*M* = 120.23, *SD* = 392.61) was detected in 126/136 samples, whereas KSHV orf74 vGPCR (*M* = 69.07, *SD* = 128.53) was detected in 127/136 samples.

**FIG. 3.**
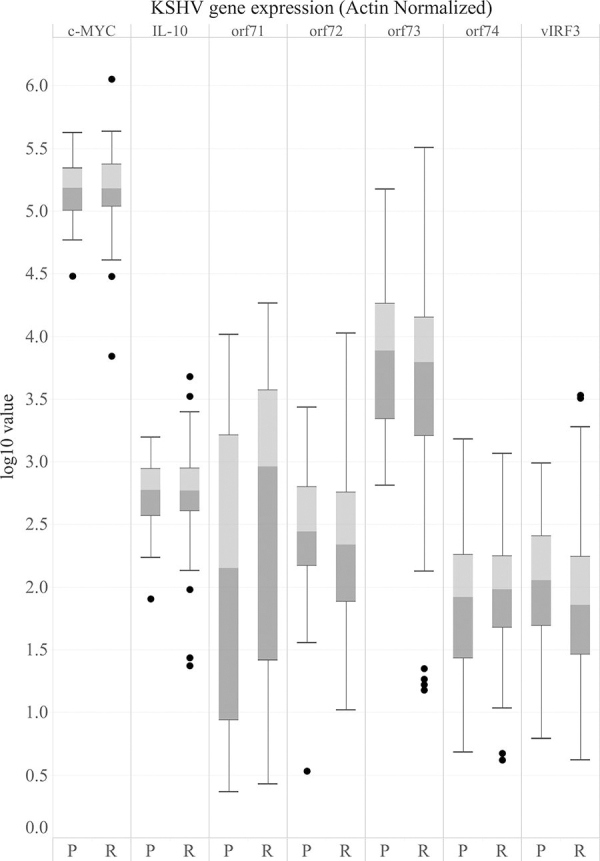
Gene expression in tumor RNA. Log10 values for each gene assayed plotted for progressors and responders, using actin-normalized data. No statistically significant trends in gene expression were found by KS progression status (*t*-test, *p* > .05).

Normalization to actin expression was used for human (e.g., *c-MYC* and *IL-10*) and KSHV genes. Human or KSHV gene expression was not associated with outcome group for the genes evaluated (Fig. 3). For KSHV genes, additional normalization to LANA expression and KSHV DNA copy number was also evaluated (Supplementary Fig. S2). Regardless of the normalization approach, the outcome group was not associated with the expression of the human or KSHV genes tested (*t*-test, *p* > .05).

### Multivariable logistic regression

**FIG. 4.**
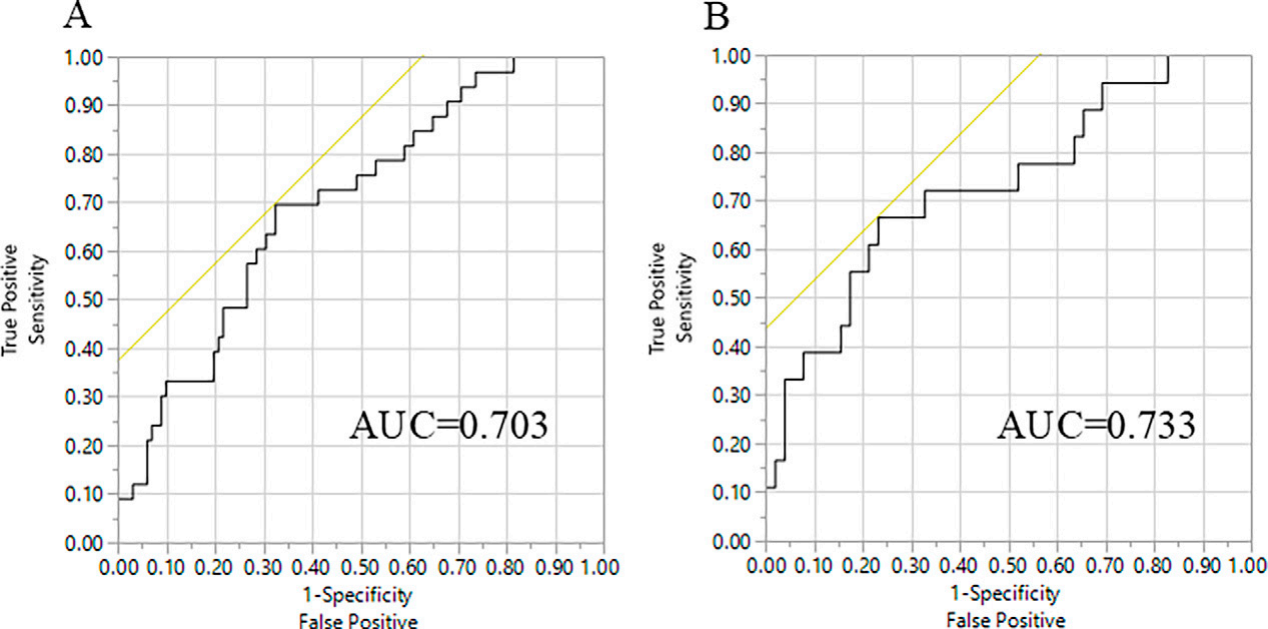
Model. **(A)** Three inputs (log10 ratio KSHV/HIV, sex and age) from all the participants in the outcomes cohort produce an AUC 0.703. **(B)** Three inputs (log10 ratio KSHV/HIV, sex, and age) from the 70 participants in the outcomes cohort with both a KSHV genotype and HIV subtype produce an AUC 0.733. AUC, area under the curve.

A series of multivariable logistic regression models were explored that used collections of patient and tumor factors as input to the model, with KS progression status as the outcome. When including all 136 participants, a model yielded reasonable performance using only the log ratio of KSHV/HIV (*p* = .013), sex (*p* = .009), and participant age (*p* = .033, AUC = 0.703) (Fig. 4). Limiting analyses to the 70 participants with both a KSHV genotype and HIV subtype assignment, the resulting model yielded quite similar results (log KSHV/HIV *p* = .020, sex *p* = .043, age *p* = .082, AUC = 0.733) (data not shown).

## Discussion

PLWH with newly diagnosed or relatively mild cases of KS generally will see dramatic improvement or remission after ART initiation without requiring additional intervention. Still, it remains unknown why, in 10%–20% of cases, KS will continue to worsen and require management with chemotherapy. Identifying biomarkers that would identify individuals at high risk for KS progression when starting ART would be potentially lifesaving, as those patients could begin chemotherapy sooner. In this study, we sought to develop a model of factors associated with KS progression after ART using baseline viral tumor measurements alone or in combination with other factors.

The outcomes cohort had a lower ratio (1.47:1) of men to women than is typically observed for KS incidence at the population level in sub-Saharan Africa (3:1).^42^ We found that men are statistically more likely than females to experience KS progression and require adjuvant chemotherapy after ART initiation, similar to a previous study.^9^ Although two other studies in sub-Saharan Africa reported that women were more likely to experience increased KS severity^42,43^ these studies were not focused on KS progression under ART. Furthermore, no statistically significant associations were found between KS progression status and baseline tumor KSHV copy number, HIV copy number, KSHV genotype, HIV subtype, KSHV gene expression, HIV plasma viral load, or CD4 count.

We also found that the logged ratio of KSHV to HIV measured in the tumor at baseline was associated with outcome. To our knowledge, we are the first group to report this finding, likely because KSHV copy number is typically measured in plasma or saliva, and HIV viral load is generally measured in plasma, rather than from the tumor as we did in this study. The increased proportion of KSHV to HIV in progressors may be linked to the number and kind of cells the two viruses infect; KSHV is found in tumor spindle cells, while HIV is generally found in immune cells. Therefore, a hypothesis for the progression of KS after ART initiation in some patients is that small numbers of local immune cells combined with higher numbers of KSHV-infected cells create an environment that promotes tumor formation and expansion. An intriguing follow-up study could combine our results with histological measurements of the ratio of tumor spindle cells to immune cells.

We measured KSHV gene expression using tumor RNA to evaluate if latent and/or lytic gene expression might be linked to KS outcomes after ART initiation. Expression of the latent genes orf73 LANA and orf72 vCyclin was found in all the samples, whereas another latent gene, orf71 v-FLIP, was not detected in 43% of the tumors, likely due to low orf71 expression and the amount of tumor RNA included in the assay. Orf71 v-FLIP has been shown to express at relatively low levels in KS tumors compared to the other latency genes^26,44^ and our results recapitulate that prior work. We detected expression of the lytic-associated genes, vIRF3 and vGPCR, in most samples, although at relatively low levels in most cases. When comparing the expression of these KSHV genes by KS progression status, we did not find a discriminating signal related to progression.

One recent study found that plasma biomarkers of the inflammation-associated CRP and the anti-inflammatory cytokine IL-10 strongly correlated with KS progression in individuals with limited-stage KS treated with ART.^11^ The authors suggested that KSHV activity might drive elevated levels of inflammatory cytokines and CRP. Our study did not find a relationship between *IL-10* levels and KSHV copy number or KSHV gene expression in the KS tumor itself, nor was there an association between tumor *IL-10* levels and progression in our cohort. This finding would indicate that the elevated plasma IL-10 protein levels associated with progression found in the other study are not due to overexpression in the tumor of *IL-10* or the KSHV genes assayed here.

Several limitations of our study should be considered. A limited quantity of tumor DNA precluded viral sequencing for some participants, so it is possible that progression-related associations may have been missed. Our sample collection did not include plasma; this measurement may have improved our model because KSHV viral load in the plasma is related to KS incidence^45,46^ and mortality.^10^ Other studies have used a battery of quantitative PCR assays to examine the expression of all KSHV genes in tumor biopsies,^44,47,48^ and other groups have used RNA-seq to profile human and KSHV gene expression simultaneously.^49–51^ We limited this study to quantitative PCR assays of the KSHV genes, as well as a few host genes, that other studies found to be important, but future studies including a less targeted approach could be performed (e.g., RNA-seq). Other studies utilized matched uninvolved skin biopsies as controls, which could also have been useful, if they were available, to compare with the tumor biopsy characteristics.

Our findings that biological sex and the log ratio of KSHV/HIV in the tumor niche are associated with KS progression are intriguing and another step toward understanding the risk factors involved in KS progression during ART. Our study included a relatively small number of participants, so future studies with more participants would be needed to confirm our findings. Ongoing work in our group is focused on assessing the tumor niche immune cell repertoire using immunohistochemistry in combination with viral load ratios. Using current methodology, identifying the copy number of KSHV to HIV in the tumor biopsy DNA requires specialized equipment and resources at considerable cost, so innovation is needed to provide simpler, lower-cost assays, which would be necessary to translate these findings into routine clinical care in resource-limited environments, where they are needed most. Given the high and persistent incidence of KS in sub-Saharan Africa, even with widespread ART availability and adherence, the demand for biomarkers to direct chemotherapy to those most likely to experience KS progression after ART administration remains imperative.

## Data Availability

Sequences were submitted to GenBank (HIV *nef* sequences PV277833-PV277964, KSHV orfK1 sequences PV289948-PV290046).
